# Comparison of glial fibrillary acidic protein-immunoglobulin G-associated myelitis with myelin oligodendrocyte glycoprotein-immunoglobulin G-associated myelitis

**DOI:** 10.3389/fneur.2023.1266067

**Published:** 2023-10-26

**Authors:** Mengyang Sun, Hao Liu, Bingqing Zhu, Yang Liu, Aijia Li, Limei Wang

**Affiliations:** ^1^Department of Neurology, The First Affiliated Hospital of Zhengzhou University, Zhengzhou, China; ^2^Zhengzhou University Medical College, Zhengzhou, China

**Keywords:** glial fibrillary acidic protein (GFAP), myelin oligodendrocyte glycoprotein (MOG), myelitis, cerebrospinal fluid, overlapping antibodies

## Abstract

**Objective:**

Glial fibrillary acidic protein-immunoglobulin G (GFAP-IgG)-associated myelitis and myelin oligodendrocyte glycoprotein-IgG (MOG-IgG)-associated myelitis have rarely been compared. Therefore, this study aimed to explore the clinical, laboratory, and imaging features of them to identify the differences.

**Methods:**

Overall, 14 and 24 patients with GFAP-IgG-and MOG-IgG-associated myelitis, respectively, were retrospectively screened and included in the study.

**Results:**

Among the 14 patients with GFAP-IgG-associated myelitis, the condition was more common in males (71.4%), with a median age of onset of 36.5 years, and more common in adults than in children (35.7%). In contrast, among the 24 patients with MOG-IgG-associated myelitis, the condition was equally divided between males and females, with a median age of onset of 9.5 years and more in children (66.7%) than in adults. The median age of onset of GFAP-IgG-associated myelitis was later than that of the MOG-IgG group. Isolated myelitis was rare in both groups. Elevated cerebrospinal fluid (CSF) protein levels were more prevalent in patients with GFAP-IgG-associated myelitis (64.3%) than in those with MOG-IgG-associated myelitis (16.7%) (*p* < 0.05), whereas patchy gadolinium enhancement of the cerebral lesion site was less common in patients with GFAP-IgG-associated myelitis than in those with MOG-IgG associated myelitis (*p* < 0.05). Six patients had a combination of other neurological autoantibodies, the specific mechanism of the overlapping antibodies remains unclear.

**Conclusion:**

Cerebrospinal fluid analysis and gadolinium enhanced MRI examination may help to distinguish the two kinds of myelitis.

## Introduction

Autoimmune glial fibrillary acidic protein astrocytopathy (GFAP-A) is an autoimmune encephalomyelitis mainly affecting the central nervous system (CNS) caused by a new autoantibody that can detect antibodies against GFAP-immunoglobulin G (GFAP-IgG) (1). The major manifestations of this disease include inflammation of the meninges, brain, spinal cord, and optic nerves (ON). Additionally, it frequently presents with a subacute onset of memory loss, confusion, one or more meningeal symptoms, and myelitis manifestations (2). Cranial imaging reveals periventricular radially oriented perivascular enhancement, and myelitis commonly manifests as longitudinally extensive transverse myelitis (LETM) (2–4). Anti-myelin oligodendrocyte glycoprotein-IgG (MOG-IgG)-associated disorders (MOGAD) are immune-mediated inflammatory demyelinating disorders of the CNS that manifest as ON, transverse myelitis, or acute disseminated encephalomyelitis (ADEM); they are usually recurrent and can lead to functional impairment due to recurrence (5, 6). MOG-IgG-associated myelitis usually presents as LETM (7). Although the clinical manifestations of myelitis usually include a combination of motor weakness, sensory symptoms, and bowel and bladder dysfunctions and are somewhat disabling, timely identifying the etiology is essential to reducing the harmful effects of inflammation (8). Furthermore, both diseases are associated with spinal cord lesions and have rarely been compared, and exploring their clinical, laboratory, and imaging features can help us understand them. Therefore, this study retrospectively analyzed and compared the clinical features of GFAP-IgG-and MOG-IgG-associated myelitis to better understand the clinical diagnosis and management processes.

## Materials and methods

### Patients

Overall, 35 and 91 patients with positive serum/cerebrospinal fluid (CSF) GFAP-IgG and MOG-IgG, respectively, who visited the First Affiliated Hospital of Zhengzhou University from October 2019 to October 2022 were retrospectively recruited, and the inclusion criteria for patients with autoimmune GFAP-A were as follows: (1) positive serum or CSF GFAP-IgG level; (2) presence of spinal cord lesions; (3) negative serum or CSF MOG-IgG and aquaporin-4-IgG (AQP4-IgG) levels; and (4) complete clinical data. In contrast, the inclusion criteria for patients with MOGAD were as follows: (1) positive serum MOG-IgG level, (2) presence of spinal cord lesions, (3) negative serum or CSF GFAP-IgG and AQP4-IgG levels, and (4) complete clinical data. Furthermore, the following were the exclusion criteria: other diseases such as traumatic brain injury, brain tumors, lead exposure, and multiple sclerosis. Based on the inclusion and exclusion criteria, 14 and 24 patients (MOG-IgG titers are presented in the Supplementary Material) with GFAP-IgG-and MOG-IgG-associated myelitis, respectively, were reported, describing their age, sex, clinical characteristics, CSF findings, brain and spinal cord magnetic resonance imaging (MRI) features, and treatment.

### Laboratory and imaging examinations

Cell-based assays were used to detect GFAP-IgG, MOG-IgG, and AQP4-IgG levels in the serum or CSF of the patients. All patients underwent spinal cord and brain imaging using 3.0 T MRI with spinal cord lesions of ≥3 and <3 segments as long- and short-segment lesions, respectively. Additionally, some patients received intravenous gadolinium injections to assess potential contrast enhancement. MRIs of the brain and spinal cord were performed by a neurologist and a neuroradiologist, respectively. Furthermore, the normal reference ranges for each laboratory index were as follows: CSF pressure: 80–180 mmH_2_O, CSF leukocytes: (0–5) × 10^6^/L, lymphocyte ratio: 60–70%, CSF protein: 150–450 mg/L, and CSF adenosine deaminase (ADA): 0–10 ng/mL.

### Ethics statement

This study followed the ethical guidelines and received ethical approval from the Ethics Committee of the First Affiliated Hospital of Zhengzhou University (2022-KY-1205-002).

### Statistical analysis

Statistical analyses and data visualization were performed using SPSS version 26.0. Normally and non-normally distributed continuous variables were expressed as mean ± standard deviation (*x* ± *s*) and median, respectively. Frequencies (percentages) were used for categorical variables. Furthermore, continuous and categorical variables were compared using the Wilcoxon rank-sum test and the chi-square or Fisher’s exact probability test, respectively. Statistical significance was considered at *p* < 0.05.

## Result

### General information

Among the 35 patients with positive CSF or serum GFAP-IgG levels, 20 and 15 were males and females, respectively. In total, 18 (51.4%) patients had spinal cord lesions, and four had overlapping antibodies. Among the 14 patients with GFAP-IgG-associated myelitis who were included, nine, two, and three were positive for GFAP-IgG in CSF, CSF and serum, and serum, respectively. Additionally, among the 91 patients with positive serum MOG-IgG levels, 47 and 44 were males and females, respectively. Thirty (33.0%) patients had spinal cord lesions, five had overlapping antibodies, and one did not undergo a CSF examination. Finally, 24 patients with MOG-IgG-associated myelitis were included, of whom 11 and 13 were positive for MOG-IgG in the serum and in both CSF and serum, respectively.

### Demographic and clinical characteristics

Among the 14 patients with GFAP-IgG-associated myelitis, 2 (14.3%) had isolated myelitis in adults. Six (42.9%) patients had a presumed or confirmed infection with prodromal symptoms; one had varicella-zoster virus (VZV) infection 2 months before the onset of neurological symptoms; two had Epstein–Barr virus (EBV) infection; one had human herpes virus type 7 infection; and two had influenza-like symptoms. Additionally, one case of combined tuberculosis and another patient with a previous diagnosis of acute Guillain-Barre syndrome 6 months earlier were identified. One patient had a history of rheumatoid arthritis and tested positive for anticyclic citrullinated peptide and rheumatoid factor. Four patients exhibited elevated levels of thyroid-associated antibodies. Furthermore, 3 (12.5%) of 24 patients with MOG-IgG-associated myelitis had isolated myelitis, all of whom were adults. In total, 10 (41.7%) patients had prodromal symptoms before the disease onset, and nine had upper respiratory tract infection symptoms, including two mycoplasma infections and one respiratory syncytial virus infection. Two cases were associated with hepatitis B virus and *Helicobacter pylori* infections. Moreover, five patients had elevated thyroid-associated antibodies; one had previous erythema multiforme and positive antinuclear antibodies; one was diagnosed with Hashimoto’s thyroiditis; and two had a history of allergic purpura. However, no tumors were found in any of the 38 patients.

**Figure 1:**
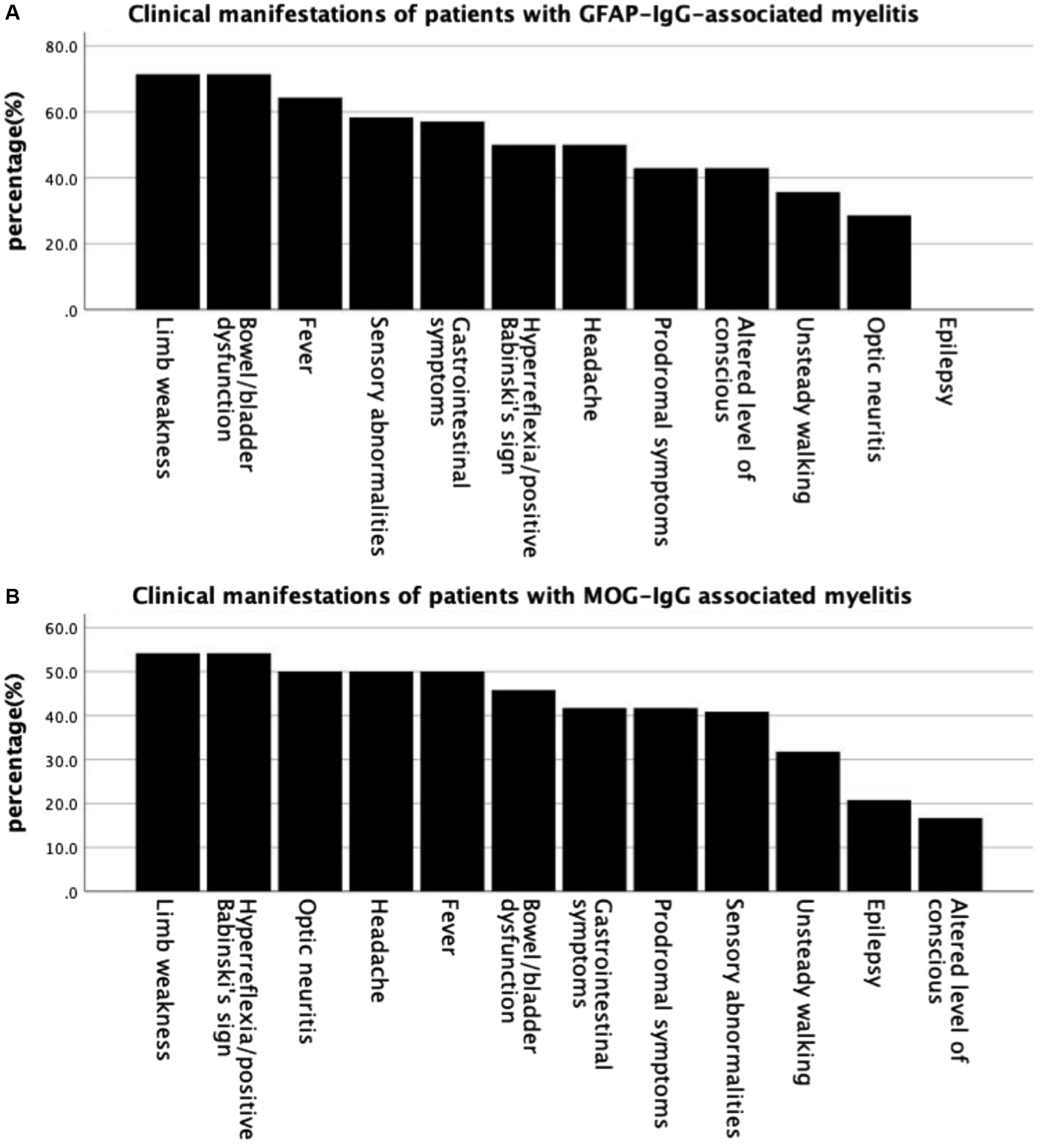
Common clinical symptoms in patients with GFAP-IgG-and MOG-IgG-associated myelitis. **(A)** Clinical manifestations of patients with GFAP-IgG associated myelitis. **(B)** Clinical manifestations of patients with MOG-IgG associated myelitis. GFAP, glial fibrillary acidic protein; IgG, immunoglobulin G; MOG, myelin oligodendrocyte glycoprotein.

Common clinical symptoms in patients with GFAP-IgG-and MOG-IgG-associated myelitis are presented in Figure 1—limb weakness is the most common in both types of myelitis. Table 1 lists the clinical characteristics of 38 patients with myelitis. Fourteen patients with GFAP-IgG-associated myelitis were more likely to be males (71.4%), with a median age of onset of 36.5 years, and more were adults than children (35.7%). In contrast, 24 patients with MOG-IgG-associated myelitis had an equal number of males and females, with a median age of onset of 9.5 years, and more were children (<18 years) (66.7%) than adults. Intravenous methylprednisolone (IVMP) was the most commonly used treatment for both myelitis types. No significant differences were found between the 14 and 24 cases of GFAP-IgG-and MOG-IgG-positive groups based on age of onset, sex ratio, proportion of children, extraspinal symptoms, symptoms/signs of myelopathy, and treatment (*p* > 0.05) (Table 1).

**Table 1:** Demographic and clinical characteristics of patients with GFAP-IgG and MOG-IgG-associated myelitis [*n* (%)].

Characteristics	GFAP-IgG, *n* = 14	MOG-IgG, *n* = 24	*p*-values
Female	4 (28.6%)	12 (50.0%)	0.309
Children	5 (35.7%)	16 (66.7%)	0.094
Age (range), years	36.5 (9–71)	9.5 (1–79)	0.060
Prodromal symptoms	6 (42.9%)	10 (41.7%)	1.000
Extraspinal symptoms			
Headache	7 (50.0%)	11/22 (50.0%)	1.000
Fever	9 (64.3%)	12 (50.0%)	0.506
Gastrointestinal symptoms	8 (57.1%)	10 (41.7%)	0.503
Optic neuritis^a^	4 (28.6%)	12 (50.0%)	0.309
Epilepsy	0 (0.0%)	5 (20.8%)	0.137
Altered level of conscious	6 (42.9%)	4 (16.7%)	0.127
Symptoms/signs of myelopathy			
Sensory abnormalities^b^	7/12 (58.3%)	9/22 (40.9%)	0.475
Bowel/bladder dysfunction	10 (71.4%)	11 (45.8%)	0.181
Hyperreflexia/positive Babinski’s sign	7 (50.0%)	13 (54.2%)	1.000
Limb weakness	10 (71.4%)	13 (54.2%)	0.329
Unsteady walking	5 (35.7%)	7/22 (31.8%)	1.000
Treatment			
Intravenous methylprednisolone	12 (85.7%)	23 (95.8%)	0.542
Intravenous immunoglobulin	5 (35.7%)	8 (33.3%)	1.000
Plasma exchange	2 (14.3%)	0 (0.0%)	0.129
Immunosuppressants	1 (7.1%)	2 (8.3%)	1.000

GFAP, glial fibrillary acidic protein; IgG, immunoglobulin G; MOG, myelin oligodendrocyte glycoprotein.

a Optic neuritis manifestations include vision loss, blurred vision, double vision, oculomotor pain or retrobulbar pain, optic papillary edema, and abnormal visual evoked potentials (VEP).

b Sensory abnormalities such as pain, painful tonic spasm, pruritus, numbness, and hypesthesia.

### CSF analysis

A CSF examination was performed in 38 patients during the acute phase, among whom elevated CSF protein levels were more prevalent in the GFAP-IgG group than in the MOG-IgG group, with significant differences (*p* < 0.05). Additionally, three patients in the GFAP-IgG group had CSF protein >1 g/L rather than in the MOG-IgG group. In both groups, patients had elevated CSF leukocyte count and lymphocyte ratio, types of oligoclonal bands (OCB), and increased CSF pressure (adults) without a significant difference (*p* > 0. 05). Furthermore, elevated CSF ADA levels were rare in both groups (Table 2).

**Table 2:** CSF findings of GFAP-IgG and MOG-IgG-associated myelitis [*n* (%)].

Findings	GFAP-IgG, *n* = 14	MOG-IgG, *n* = 24	*p*-values
Elevated leukocyte	12 (85.7%)	20 (83.3%)	1.000
Significantly elevated leukocyte^a^	8 (57.1%)	9 (37.5%)	0.318
Elevated protein	9 (64.3%)	4 (16.7%)	0.005
Elevated lymphocyte ratio	12 (85.7%)	14 (58.3%)	0.147
Elevated pressure^b^	3/9 (33.3%)	2/8 (25.0%)	1.000
OCB in CSF (type 2)	2 (14.3%)	7 (29.2%)	0.438
OCB in CSF and serum (type 3)	2 (14.3%)	1 (4.2%)	0.542
Elevated ADA	1/13 (7.7%)	0/15 (0.0%)	0.464

OCB, oligoclonal band; CSF, cerebrospinal fluid; ADA, adenosine deaminase; GFAP, glial fibrillary acidic protein; IgG, immunoglobulin G; MOG, myelin oligodendrocyte glycoprotein.

a Significantly elevated leukocyte count >50 × 10*^6^/L.

b Elevated pressure: >180 mm H_2_O. (The pressure values in this table are for adult patients only).

### Imaging characteristics

**Figure 2:**
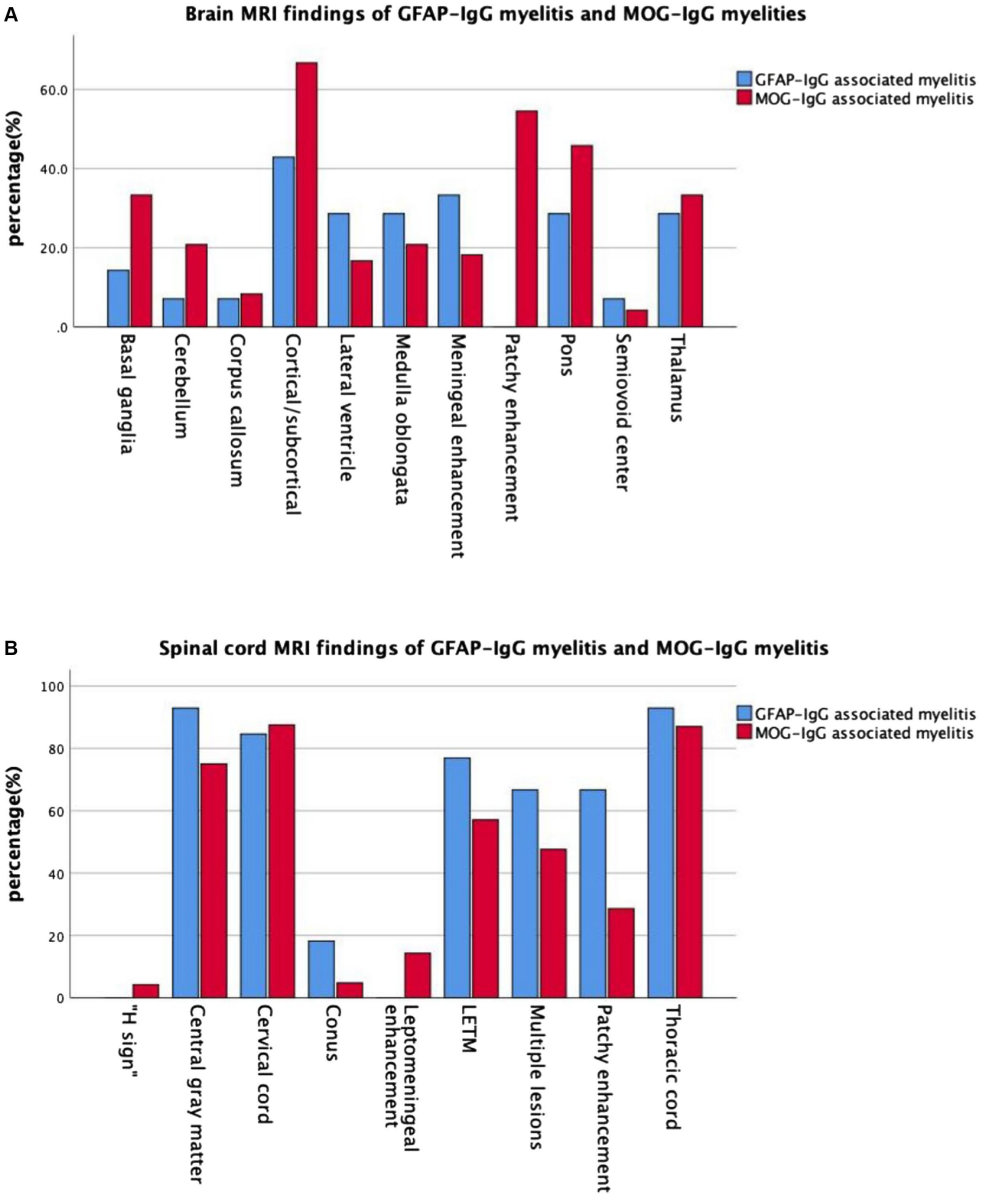
Imaging manifestations in the two groups of patients with myelitis. **(A)** Brain MRI and **(B)** spinal MRI manifestations in the two groups of patients with myelitis. MRI, magnetic resonance imaging; GFAP, glial fibrillary acidic protein; IgG, immunoglobulin G; MOG, myelin oligodendrocyte glycoprotein.

**Figure 3:**
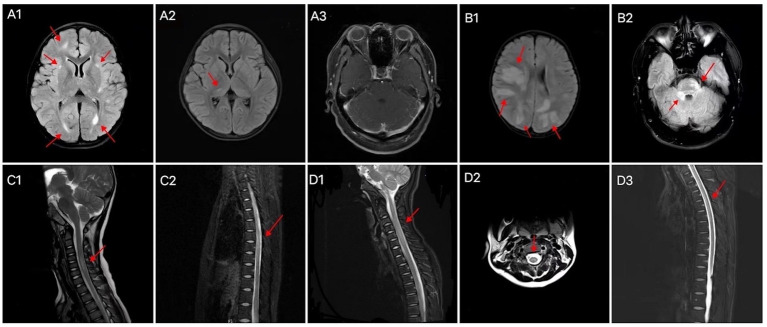
Brain MRI characteristics of patients with **(A)** GFAP-IgG-and **(B)** MOG-IgG-associated myelitis. Abnormal signals in the right frontal lobe, bilateral lateral posterior horn of the ventricle, and bilateral basal ganglia areas **(A1)**; abnormal signals in the right thalamus **(A2)**; bilateral cerebellar multiple strip enhancement **(A3)**; multiple abnormal signals in the cortex/subcortex **(B1)**; abnormal signal in the right pontocerebellar commissure and pons **(B2)**. Spinal cord MRI characteristics of patients with GFAP-IgG-associated myelitis (**C1, C2**) and MOG-IgG-associated myelitis (**D1–D3**). LETM of the cervical cord **(C1)**, LETM of the thoracic cord **(C2)**, LETM of the cervical cord **(D1)**, “H sign” **(D2)**, and LETM of the thoracic cord **(D3)**. LETM, longitudinally extensive transverse myelitis; MRI, magnetic resonance imaging; GFAP, glial fibrillary acidic protein; IgG, immunoglobulin G; MOG, myelin oligodendrocyte glycoprotein.

Cranial and spinal MRIs were performed in 38 patients. We depicted the two patient groups’ imaging performance to visualize their differences (Figure 2). Among the 14 patients with GFAP-IgG-associated myelitis, 11 (78.6%) had intracranial lesions, which mainly manifested as high signals on T2 weighted image/fluid-attenuated inversion recovery (T2WI/FLAIR) sequences (Figures 3A1,A2). Nine patients underwent gadolinium enhancement scans, of whom three and two had meningeal and cerebellar enhancements, respectively (Figures 3A3). LETM was present in 10 cases (76.9%) (Figures 3C1,C2), and 13 cases (92.9%) had the involvement of central gray matter. Additionally, 21 of 24 patients (87.5%) with MOG-IgG-associated myelitis had intracranial lesions, which were characterized by abnormal signals on T2WI/FLAIR (Figures 3B1,B2). Eleven cranial gadolinium-enhanced scans were performed, 6 (54.5%) showed patchy enhancement, 12 (57.1%) with LETM (Figures 3D1,D3), and 18 (75.0%) with involvement of the central gray matter (1 case was confined to the gray matter of the spinal cord, with an axial appearance of the “H sign”; Figures 3D2). In cranial MRI, patchy gadolinium enhancement was less common in patients with GFAP-IgG-associated myelitis than in those with MOG-IgG-associated myelitis, with a significant difference (*p* < 0.05). No significant differences were found between the two groups regarding cranial MRI lesion site, spinal cord long-segment lesions, lesion distribution, or site of involvement (*p* > 0. 05) (Table 3).

**Table 3:** MRI characteristics of GFAP-IgG and MOG-IgG-associated myelitis [*n* (%)].

Characteristics	GFAP-IgG, *n* = 14	MOG-IgG, *n* = 24	*p*-values
Intracranial lesion	11 (78.6%)	21 (87.5%)	0.650
Meningeal enhancement	3/9 (33.3%)	2/11 (18.2%)	0.617
Patchy enhancement	0/9 (0.0%)	6/11 (54.5%)	0.014
Cortical/subcortical	6 (42.9%)	16 (66.7%)	0.187
Medulla oblongata	4 (28.6%)	5 (20.8%)	0.699
Lateral ventricle	4 (28.6%)	4 (16.7%)	0.433
Thalamus	4 (28.6%)	8 (33.3%)	1.000
Pons	4 (28.6%)	11 (45.8%)	0.329
Basal ganglia	2 (14.3%)	8 (33.3%)	0.268
Corpus callosum	1 (7.1%)	2 (8.3%)	1.000
Semiovoid center	1 (7.1%)	1 (4.2%)	1.000
Cerebellum	1 (7.1%)	5 (20.8%)	0.383
Spinal cord MRI			
LETM	10/13 (76.9%)	12/21 (57.1%)	0.292
Cervical cord	11/13 (84.6%)	21 (87.5%)	1.000
Thoracic cord	13 (92.9%)	20/23 (87.0%)	1.000
Conus	2/11 (18.2%)	1/21 (4.8%)	0.266
Central gray matter	13 (92.9%)	18 (75.0%)	0.227
“H sign”*	0 (0.0%)	1 (4.2%)	1.000
Multiple lesions	8/12 (66.7%)	10/21 (47.6%)	0.469
Leptomeningeal enhancement	0/6 (0.0%)	1/7 (14.3%)	1.000
Patchy enhancement	4/6 (66.7%)	2/7 (28.6%)	0.286

*Spinal cord MRI T2-hyperintensity is confined to gray matter on axial sequences, forming the “H sign.” LETM, longitudinally extensive transverse myelitis; MRI, magnetic resonance imaging; GFAP, glial fibrillary acidic protein; IgG, immunoglobulin G; MOG, myelin oligodendrocyte glycoprotein.

### Overlapping antibodies

In addition to the 38 patients included, six with myelitis had other neurological autoantibodies in combination (Table 4). Patient 1 was admitted to the hospital with weakness in the limbs and unsteady walking and was administered IVMP, intravenous immunoglobulin (IVIG), and plasma exchange (PE). She was discharged from the hospital with improved symptoms compared to the previous visit. Two months later, she was revisited, and the relevant antibodies turned negative. Moreover, no obvious discomfort was noted. Patient 2 was admitted to the hospital because of vomiting and unsteady walking, and the test results suggested an EBV infection. Additionally, a vision examination suggested decreased vision in both eyes and abnormal visual evoked potential (VEP) in the right eye. The patient was administered IVMP, IVIG, and immunosuppressive treatments and was reexamined with a negative GFAP-IgG level. Her symptoms improved, and she was discharged from the hospital with mycophenolate mofetil (MMF) and a small dose of steroid. Subsequently, the patient experienced recurrence and left-side blindness in the right eye. Patient 3 was admitted to the hospital with blurred vision in the right eye and numbness and weakness in both lower limbs. The patient had previous symptoms of an antecedent infection and was treated with IVMP, IVIG, and MMF. He had three recurrences, and the antibody was negative on follow-up. The patient’s muscle strength was better than before, although he walked unsteadily. Patient 4 was admitted to the hospital with blurred vision in the right eye. She had a fever, dizziness, and drowsiness 1 month prior. The patient was treated with IVMP and was discharged from the hospital after her vision improved. Subsequently, she was treated with low-dose steroid and reviewed regularly. The patient had blurred vision in the left eye and was discharged from the hospital after being treated with MMF. She relapsed several times and used other immunosuppressive agents; however, vision loss persisted in both eyes. Patient 5 was admitted to the hospital with blurred vision in the right eye and experienced headaches, fever, and seizures 20 days before the disease’s onset. A physical examination revealed sensory abnormalities in both lower limbs. After admission, the patient had intermittent speech disorganization and was treated with IVMP and IVIG. He was discharged from the hospital after his psychiatric symptoms had improved and was readmitted with diplopia 3 months later. Finally, he continued receiving IVMP and was administered small doses of steroid and MMF outside the hospital. Patient 6 was admitted to the hospital with intermittent seizures and fever, and laboratory tests suggested EBV infection, blurred vision, IVMP treatment, and improved vision. Finally, she was discharged for convulsive episodes with lethargy and dysuria.

**Table 4:** Clinical, CSF, and imaging characteristics of patients with overlapping antibodies.

Case number /sex/age, y	Clinical symptoms	Antibody (CSF)	Antibody (serum)	CSF leukocytes	CSF protein	MRI	Treatment
NO.1 Female 11	Weakness of both lower limbs, unstable walking, drowsiness, loss of appetite, vomiting, urinary and bowel disorders, seizure, hallucinations, and change in temperament	GFAP MOG NMDAR	GFAP MOG NMDAR	18 × 10^6^/L	262.0 mg/L	Frontal lobe, C3-5, T10	IVMP, IVIG, and PE
NO.2 Female 12	Vomiting, unsteady walking, blurred vision, fever, itchy skin, crooked mouth, and incomplete eyelid closure	GFAP AQP4	AQP4	11 × 10^6^/L	443.0 mg/L	Pontine brain medulla oblongata C1–5, T1, T7, T10–11	IVMP, IVIG, and immunosuppressants
NO.3 Male 36	Blurred vision in the right eye, numbness and weakness in both lower limbs, episodic limb spasms and convulsions, fever, unsteady walking, and bowel/bladder dysfunction	GFAP MOG	MOG	38 × 10^6^/L	342.0 mg/L	Frontal lobe, thalamus, cerebral peduncle, periventricular area, cerebellar, C1–T3	IVMP, IVIG, and immunosuppressants
NO.4 Female 4	Blurred vision, fever, and itchy skin	MOG	GFAP MOG	22 × 10^6^/L	407.0 mg/L	C4-T8	IVMP and immunosuppressants
NO.5 Male 26	Visual confusion, fever, headache, seizure, dizziness, abnormal sensation in both lower limbs, and intermittent speech confusion	NMDAR	MOG NMDAR	40 × 10^6^/L	342.0 mg/L	Cortical/subcortical, pons, pontine arms, cerebral peduncles, and thalamus C5-T10	IVMP and IVIG
NO.6 Female 6	Seizures, fever, tremors, headache, loss of vision, drowsiness, and urinary disturbances	MOG	MOG IgLON5	71 × 10^6^/L	340.0 mg/L	Optic nerve C3-T1	IVMP

C, cervical spinal cord; T, thoracic spinal cord; IVMP, intravenous methylprednisolone; IVIG, intravenous immunoglobulin; PE, plasma exchange; MRI, magnetic resonance imaging; NMDAR, N-methyl-d-aspartate receptor; GFAP, glial fibrillary acidic protein; MOG, myelin oligodendrocyte glycoprotein; AQP4, aquaporin-4; CSF, cerebrospinal fluid.

## Discussion

This study retrospectively compared the clinical features, CSF and imaging features, and overlapping antibodies in 14 and 24 patients with GFAP-IgG-and MOG-IgG-associated myelitis, respectively.

In our study, GFAP-IgG-associated myelitis was prevalent in adults, consistent with the results of previous studies (4, 9, 10). Two patients (14.3%) had isolated myelitis, and all children had a combined presentation of encephalitis. Four patients (28.6%) were females, while 4 (31%) were females in a previous study (9). However, other studies reported a female majority (4, 10). There are few studies on myelitis; the number of cases is small, and there is some variation in the findings. Therefore, a large-scale study is required to further confirm whether there are sex differences. Patients with prodromal symptoms in GFAP-IgG-associated myelitis are relatively common (42.9% in our study), viral infections are common, and some studies have shown that autoimmune GFAP-A may be associated with herpes simplex virus infection (3, 11, 12). One patient in our study had a VZV infection 2 months before the disease onset; Dubey et al. (13) reported one patient who developed autoimmune GFAP-A a few weeks after VZV encephalitis. Therefore, further studies are needed to determine whether this disease is associated with viral infections. In contrast, compared to previous studies [16/54 (30%)] (14), MOG-IgG-associated myelitis was more prevalent in children (66.7%). In a study of 54 patients with MOG-IgG-associated myelitis, 24 (44%) were females (14), while 15 (39.5%) were females in another study (15). In our study, 12 (50%) patients were female. No significant sex differences were observed between groups. Although 41.8% of patients with MOG-IgG-associated myelitis have a prodromal event, such as infection or vaccination, before disease onset (15), our study revealed such cases in 41.7% of patients; however, no vaccination was found. Four patients had other autoimmune diseases, suggesting that the appearance of MOGAD may be related to an immune disorder. A Chinese study showed that 29.2% (38/130) of patients with MOGAD had myelitis (15). Myelitis is the second most common manifestation of MOGAD in adults, accounting for 18–52% of cases (16). In our study, 30 patients (33.0%) had spinal cord lesions on MRI with a younger age of onset. Limb weakness, dysuria, and sensory abnormalities were common in the GFAP-IgG group, which could also have manifested as unsteady walking (35.7%). Xu et al. (4) showed that 1 of 19 patients exhibited unsteady walking and dysuria. Furthermore, in our study, ON symptoms were relatively uncommon in the GFAP-IgG group than MOG-IgG group, and no epilepsy was found, nor were any of the patients in the study by Fang et al. (1) presented with epilepsy. Nine (69%) patients in the study by Sechi et al. (9) presented with tremors, which were not observed in our patients. Moreover, the 38 patients included showed that most of them with myelitis in both groups also had extraspinal symptoms, and nonspecific symptoms, such as headache and fever, were common in both groups. In both groups, the treatment was generally IVMP. Therefore, compared to MOG-IgG-associated myelitis, GFAP-IgG-associated myelitis appears to have a later age of onset, is relatively rare in patients with optic neuritis, and is relatively common in those with impaired consciousness.

CSF findings in both groups showed inflammatory changes and a predominance of elevated lymphocytes, and OCB was observed in both the serum and CSF. In our study, 12 (85.7%) and 9 (64.3%) patients with GFAP-IgG-associated myelitis had elevated CSF leukocytes and CSF proteins, respectively, and all 13 patients in a previous study had increased CSF leukocytes (9). In another retrospective study of 16 patients with GFAP-IgG-associated myelitis, the median CSF protein concentration was 729 mg/L and approximately 2,344 mg/L (10). Our CSF protein was approximately 1837 mg/L, including three cases >1 g/L; therefore, we hypothesize that protein elevation is more pronounced in the CSF of patients with GFAP-IgG-associated myelitis. However, future sample sizes should be expanded to provide more evidence. Most of the patients (83.3%) in the MOG-IgG group had elevated CSF leukocytes, with nine (37.5%) having >50 × 10^6^/L; other studies have shown that 45–55% of patients can have significantly elevated CSF leukocytes (14, 17). A study reported elevated CSF protein levels in 77% of the 35 patients with MOG-IgG-associated myelitis (18). Contrary to our study (16.7%), maybe the elevation of CSF proteins was not significant in pediatric patients with MOGAD compared to adults. No significant elevation in CSF ADA levels was observed in either group. Previous studies have suggested that most patients with autoimmune GFAP-A show a transient elevation in CSF ADA levels during the first month of onset (19). Therefore, we hypothesized that GFAP-IgG-associated myelitis is more likely to be associated with elevated CSF protein levels and is more significantly increased than MOG-IgG-associated myelitis, which may indicate a severe inflammatory response in the CNS.

Autoimmune GFAP-A has been shown to exhibit typical periventricular radially oriented perivascular enhancement in approximately 40–50% of cases (1–3, 20). While intracranial lesions in patients with GFAP-IgG-associated myelitis in our study were found in the cortical/subcortical, medulla oblongata, lateral ventricles, thalamus, pons, and basal ganglia, among others, nine of them had cranial MRI enhancement scans, although this typical enhancement was not observed. However, three cases (33.3%) were abnormal and showed meningeal enhancement, and cerebellar meningeal enhancement was observed in two cases. In a Chinese cohort, cranial MRI of this disease was extremely rare in patients with periventricular radially oriented perivascular enhancement, whereas cerebellar meningeal enhancement was noteworthy (21). In another study, no cases of this typical enhancement were observed (22). Intracranial lesions in MOGAD tend to present as periventricular lesions extending from nearby cortical lesions with large lesion areas. Nodular enhancement has been observed on enhancement scans (23, 24). Eleven of our patients with MOG-IgG-associated myelitis underwent cranial enhancement scans, and six (54.5%) showed patchy enhancement. In our study, LETM was common in patients with GFAP-IgG-associated myelitis. Additionally, the cervical and thoracic spinal cords were susceptible; 2/11 (18.2%) involved the conus, >90% involved the central gray matter, and multiple lesions were common, consistent with a report of 19 patients by a Chinese author (4). One study reported that the spinal cord central canal, punctate, or leptomeningeal enhancement is reportedly typical of GFAP-IgG-associated myelitis (9). Among the six patients with gadolinium enhancement in this study, four (66.7%) showed patchy enhancement. None of our 14 patients with GFAP-IgG myelitis had spinal cord lesions confined to gray matter on axial MRI sequences (expressed as the “H sign”). LETM and short-segment lesions were found in 12 (57.1%) and 9 (42.9%) patients with MOG-IgG-associated myelitis, similar to previous reports, which showed that LETM is the predominant pattern in MOG-IgG-associated myelitis; however, short-segment lesions are also common (16, 17). MRI lesions of the spinal cord in patients with MOG-IgG-associated myelitis, predominantly involving the central gray matter, were limited to the axial sequence of only one patient (4.2%) exhibiting a more pronounced “H sign,” which differs from the findings of previous studies (14). A Chinese cohort study showed that only 3 out of 29 (10.3%) patients with MOGAD had spinal cord enhancement that was limited to gray matter (24). Our study showed that patients with MOG-IgG-associated myelitis frequently had involvement of the cervical and thoracic spinal cords, while conus involvement was rare. Another study showed that myelitis was common in the thoracic and lumbar spinal cord (25), and conus involvement may have been relatively rare in the Chinese cohort. Cerebellar meningeal enhancement appeared to occur more frequently in patients with GFAP-IgG-associated myelitis. Based on a previous study (14), the “H sign” of spinal MRI, which is confined to the gray matter, may also be an imaging feature that distinguishes the two types of myelitis.

Overlapping antibodies were observed for both autoimmune GFAP-A and MOGAD (26). Six patients in our study with a combination of other neurological autoantibodies had a younger age of onset (maximum age, 35 years). Three (50%) patients had prodromal symptoms or co-infection with a viral infection; however, no tumors were detected. Patients with autoimmune encephalitis (AE) usually present with rapidly progressive cognitive dysfunction, refractory epilepsy, and psychiatric abnormalities (27). In our study, all three patients with positive autoimmune antibodies showed related symptoms (patients 1, 5, and 6). Therefore, patients with overlapping antibodies can have both disease manifestations, such as symptoms of AE and demyelinating disease, which may complicate the diagnosis. Whether patients with overlapping antibodies are more likely to have tumors remains unclear; however, no tumors were found in the six patients in this study, allowing for long-term follow-up. Moreover, the specific mechanism of the overlap syndrome has not been fully elucidated; therefore, more studies are needed to further elucidate the underlying mechanism.

MOG is a glycoprotein that is specifically expressed in CNS oligodendrocytes, and anti-MOG-IgG is a pathogenic antibody against MOGAD (28). Although GFAP is an intracellular protein, antibodies against intracellular antigens are unlikely to reach their targets *in vivo* and are not usually considered pathogenic, and a T-cell-mediated inflammatory response is considered the primary mechanism of neuronal destruction (29). Some pathological biopsies support a CD8+ T-cell-mediated autoimmune response (30). However, the pathogenesis of these diseases remains unclear, and more research is needed to elucidate them and better understand and treat them.

## Limitations

First, there may be bias due to the retrospective design of the study, as well as a case selection bias because of its single-center nature. Therefore, future prospective cohort studies with larger sample sizes should further clarify the differences between the two groups. Second, the specificity was low when only serum GFAP-IgG was positive, and 3 of 14 GFAP-IgG myelitis cases were only serum positive; however, all cases had characteristic clinical syndromes, and other diseases were reasonably excluded. Lastly, this study focused on comparing clinical features between the two groups of patients rather than on clinical outcomes or long-term follow-up.

## Conclusion

This study provides clinical, CSF, and MRI evidence for recognizing and differentiating GFAP-IgG-and MOG-IgG-associated myelitis. Therefore, these findings will help clinicians better recognize these diseases.

## Data availability statement

The raw data supporting the conclusions of this article will be made available by the authors, without undue reservation.

## Ethics statement

The studies involving humans were approved by the Ethics Committee of the First Affiliated Hospital of Zhengzhou University (2022-KY-1205-002). The studies were conducted in accordance with the local legislation and institutional requirements. Written informed consent for participation in this study was provided by the participants’ legal guardians/next of kin.

## Author contributions

MS: Writing – original draft, Data curation, Formal analysis. HL: Data curation, Formal analysis, Writing – review & editing. BZ: Data curation, Formal analysis, Writing – review & editing. YL: Data curation, Formal analysis, Writing – review & editing. AL: Data curation, Formal analysis, Writing – review & editing. LW: Writing – review & editing.
