# Supplementary material for: Comparison of glial fibrillary acidic protein-immunoglobulin G-associated myelitis with myelin oligodendrocyte glycoprotein-immunoglobulin G-associated myelitis
Source: Front Neurol. 2023 Oct 26;14:1266067. doi: 10.3389/fneur.2023.1266067 (PMC10645061; doi:10.3389/fneur.2023.1266067)
Supplement: Supplementary file 1 [file Data_Sheet_1.docx]

***Supplementary Material***

Serum MOG-IgG titers of 24 patients with MOG-IgG associated myelitis

| Case number | MOG-IgG titers | Additional supporting features required |
| --- | --- | --- |
| NO.1 | 1:32 | AQP4-IgG seronegative; Longitudinally extensive myelitis; T2-hyperintensity involving pons |
| NO.2 | 1:100 |  |
| NO.3 | 1:320 |  |
| NO.4 | 1:32 | AQP4-IgG seronegative; Longitudinally extensive myelitis; T2-hyperintensity involving pons |
| NO.5 | 1:100 |  |
| NO.6 | 1:32 | AQP4-IgG seronegative; Longitudinally extensive myelitis; Optic neuritis：bilateral simultaneous clinical involvement |
| NO.7 | 1:320 |  |
| NO.8 | 1:100 |  |
| NO.9 | 1:10 | AQP4-IgG seronegative; Multiple ill-defined T2 hyperintense lesions in supratentorial and often infratentorial white matter |
| NO.10 | 1:320 |  |
| NO.11 | Positive | AQP4-IgG seronegative; Optic disc oedema; Optic neuritis：bilateral simultaneous clinical involvement |
| NO.12 | 1:10+ | AQP4-IgG seronegative; Optic neuritis：bilateral simultaneous clinical involvement; Multiple ill-defined T2 hyperintense lesions in supratentorial and often infratentorial white matter |
| NO.13 | 1:100 |  |
| NO.14 | 1:32 | AQP4-IgG seronegative; Longitudinally extensive myelitis |
| NO.15 | 1:1000+ |  |
| NO.16 | 1:100 |  |
| NO.17 | 1:32 | AQP4-IgG seronegative; Bilateral simultaneous clinical involvement |
| NO.18 | 1:100 |  |
| NO.19 | 1:320 |  |
| NO.20 | 1:32 | AQP4-IgG seronegative; Multiple ill-defined T2 hyperintense lesions in supratentorial and often infratentorial white matter |
| NO.21 | 1:100 |  |
| NO.22 | Positive | AQP4-IgG seronegative; Longitudinally extensive myelitis |
| NO.23 | Positive | AQP4-IgG seronegative; Longitudinally extensive myelitis |
| NO.24 | Positive | AQP4-IgG seronegative; T2-hyperintensity involving pons; Bilateral simultaneous clinical involvement |
